# A Simple Linear-Type Negative Permittivity Metamaterials Substrate Microstrip Patch Antenna

**DOI:** 10.3390/ma14164398

**Published:** 2021-08-06

**Authors:** Wei-Hua Hui, Yao Guo, Xiao-Peng Zhao

**Affiliations:** Department of Applied Physics, School of Physical Science and Technology, Northwestern Polytechnical University, Xi’an 710129, China; huiweihua1118@mail.nwpu.edu.cn (W.-H.H.); guoyao2012@mail.nwpu.edu.cn (Y.G.)

**Keywords:** metamaterials, negative permittivity, microstrip patch antenna, gain

## Abstract

A microstrip patch antenna (MPA) loaded with linear-type negative permittivity metamaterials (NPMMs) is designed. The simple linear-type metamaterials have negative permittivity at 1–10 GHz. Four groups of antennas at different frequency bands are simulated in order to study the effect of linear-type NPMMs on MPA. The antennas working at 5.0 GHz are processed and measured. The measured results illustrate that the gain is enhanced by 2.12 dB, the H-plane half-power beam width (HPBW) is converged by 14°, and the effective area is increased by 62.5%. It can be concluded from the simulation and measurements that the linear-type metamaterials loaded on the substrate of MAP can suppress surface waves and increase forward radiation well.

## 1. Introduction

Electromagnetic (EM) metamaterials are composed of periodic, subwavelength artificial structures, which can be resonant or non-resonant units. A two-dimensional form of metamaterials is called a metasurface. According to the uniform effective medium theory, the properties of these structures can be determined by the effective permittivity and permeability. When an EM wave is incident on the metamaterials, they often show abnormal physical properties that natural materials do not possess, such as near zero refraction, negative permittivity, negative permeability, or double-negative. Metamaterials with a single negative property can suppress the propagation of EM waves. Metamaterials with double negative properties are called left-handed metamaterials (LHMs), obeying the left-hand rule. Veselago firstly proposed the concept of metamaterials in 1968 [1]. Pendy et al. proved the existence of left-handed metamaterials in the microwave frequency range in 2001. Since then, metamaterials have attracted a lot of attention in many fields, such as EM stealth cloak [2], perfect absorber [3,4,5], EM energy harvest [6,7,8,9], sensor, and polarization devices [10,11,12].

Microstrip patch antenna (MPA) has many advantages of light weight, small size, easy processing, low profile, and easy to make dual-band or circular polarization antenna. However, MPA also has some disadvantages including narrow bandwidth, low efficiency, low gain, and narrow 3 dB axial ratio bandwidth, which restrict its integration with modern multifunctional wireless devices [13].

In order to overcome these disadvantages of MPA, researchers have adopted metamaterials with different EM properties to refine its performances [14,15,16,17,18]. Nasimuddin et al. embedded two-dimensional rectangular-ring units into the substrate of a circularly polarized patch antenna, so that the VSWR bandwidth of the antenna reached 35.6%, the 3 dB axis ratio is increased by a factor of seven, and the gain is increased by about 3 dB [14]. Singh et al. subsequently proposed a Fabry–Perot resonator structure MPA based on a double annular slot resonator unit cell superstrate, which increases the gain by 12.5 dB [15]. Gao et al. proposed a three-layer high refractive index metamaterial (HRIM) superstrate to enhance the performance of MPA. The gain and bandwidth are enhanced greatly [17]. Then, Borazjani et al. proposed a high-gain broadband MPA composed of four layers of negative refractive index metamaterials superstrate, and the gain is increased by about 10 dB [18]. Although these antennas have been modified notably by adopting various metamaterial superstrates, the high profile limits their practical application.

In order to solve the limitation of these antennas’ high profile, our team used metamaterials with negative permeability as the substrate of the MPA to suppress surface waves and successfully improved the performance of MPA [19,20]. However, the effective bandwidth of negative permeability metamaterials is too narrow, which is only suitable for these antennas with a narrow bandwidth. Subsequently, Zhou et al. adopted a meander-line unit with near-zero permittivity to enhance the directivity of the Vivaldi antenna [21]. Yang et al. employed artificial magnetic conductors (AMCs) under the edge-fed patch antenna to improve the bandwidth and gain [22]. Cao et al. applied parasitic patches around the radiation patch on the substrate, and the effective bandwidth of the parasitic patches is close to 2 GHz [23]. Therefore, it is still a challenge to adopt single metamaterials to improve the EM performance of an ultra-wideband (UWB) or multi-band microstrip antennas. The metal wire with electrical continuity has negative permittivity in a wider band, which has great potential in the UWB antenna or multi-band antenna array.

In this paper, in order to form an MPA with a negative permittivity substrate, simple linear-type metamaterials units are loaded around the center patch in an annular manner. Four patch antennas loaded with negative permittivity metamaterials (NPMMs) are simulated at the L-band, S-band, X-band, and C-band, respectively. A group of X-band antennas are fabricated and measured. The simulated and experimental results consistently reveal that the linear-type NPMMs can improve the performance of MPA effectively in a wide band.

## 2. Design of Negative Permittivity Metamaterials (NPMMs)

Pendry shows that the effective permittivity of the cut-wire medium can be expressed as [24,25]:

(1) $$\varepsilon \left(\omega \right)=1-\frac{\omega_{p}^{2}-\omega_{0}^{2}}{\omega^{2}-\omega_{0}^{2}+i\omega \Gamma }$$

where $\omega_{p}$ is the plasma frequency, $\omega_{0}$ is the resonance frequency, which is determined only by the geometry of wire and is independent of the charge, effective mass, and density of electrons, and Γ is system loss. For $\omega_{0}<\omega <\omega_{p}$, the permittivity is negative, and there is no EM wave propagation mode in the structure. The cross-section of the metal wire obtained by the printed circuit board is rectangular, and its plasma frequency can be expressed as:

(2) $$f_{p}=\frac{c_{0}}{g\sqrt{2\pi \ln\left(g/\sqrt{\mathrm{mt}/\pi }\right)}}$$

In this equation, g  is the lattice constant, $c_{0}\;$ is the speed of light in vacuum, m is the width of the metal line, and t is the thickness of the metal line. When the metal line metamaterials have electrical continuity, the resonance frequency $\omega_{0}\;$ is zero.

**Figure 1:**
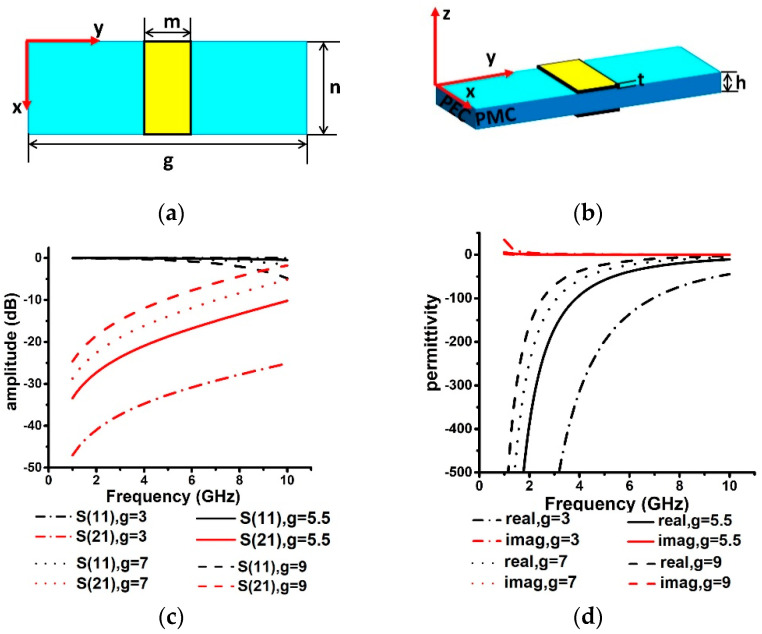
Structure and EM properties of linear-type metamaterials unit: (**a**) top view; (**b**) side view; (**c**) S11 and S21; (**d**) permittivity.

Based on the above theory, the unit of the linear-type metamaterials is shown in Figure 1a,b. The structure of the metamaterial is very simple, consisting of a substrate and double-sided etched metal wires. The substrate is a rectangular polytetrafluoroethylene (PTFE)$\;(\varepsilon_{r}=3.5$, tanδ=0.001). The structural parameters are shown as the following: n = 1 mm, g = 5.5 mm, m = 0.5 mm, t = 0.035 mm, h = 2 mm. Then, these parameters are introduced into Equation (2) to calculate the plasma frequency. The plasma frequency $f_{p}\;$ can be obtained as 10.49 GHz.

The simulation software high-frequency structure simulator (HFSS) is employed to optimize the linear-type metamaterials. The software uses the finite element method to solve the EM model. The electronic boundaries are parallel to the x-axis, and the magnetic boundaries are parallel to the y-axis. The solution frequency is 10 GHz, the solver accuracy is 0.001, and the maximum number of passes is 20. The simulated reflection and transmission curves of the linear-type metamaterials are shown in Figure 1c. It means that the transmission forbidden band is from 1 to 10 GHz, and EM waves cannot pass through while the metamaterials unit interval g is 3 mm, 5.5 mm, 7 mm, and 9 mm.

In the study of metamaterials, it is generally described as effective medium theory. When the wavelength is much larger than the size of the structure, the electromagnetic wave will not identify its internal structure, so that the properties of the metamaterials can be described by effective permittivity and effective permeability. According to the above theory, the permittivity of linear-type metamaterials can be calculated by the scattering parameter extraction method [26,27]. The electromagnetic wave is a perpendicular incident to the linear-type metamaterials. The transmission and reflection can be obtained by simulation. The refractive index n and wave impedance z can be obtained by the transmission matrix and the S-parameter, and the effective permittivity ε and permeability μ can be further obtained by Equations (5) and (6).

(3) $$n=\frac{1}{\mathrm{kd}}\cos\left[\frac{1}{2S_{21}}\left(1-S_{11}{}^{2}+S_{21}{}^{2}\right)\right]$$

(4) $$z=\pm \sqrt{\frac{{\left(1+S_{11}\right)}^{2}-S_{21}{}^{2}}{{\left(1-S_{11}\right)}^{2}-S_{21}{}^{2}}}$$

(5) $$\varepsilon =\frac{n}{z}$$

(6) μ=nz

The real and imaginary parts of the permittivity calculated by the scattering parameter extraction method are shown in Figure 1d. It denotes that the real part of the permittivity is negative from 1 to 10 GHz. This is basically consistent with the result calculated according to Equation (2).

## 3. Microstrip Patch Antenna Loaded with Negative Permittivity Metamaterials

**Figure 2:**
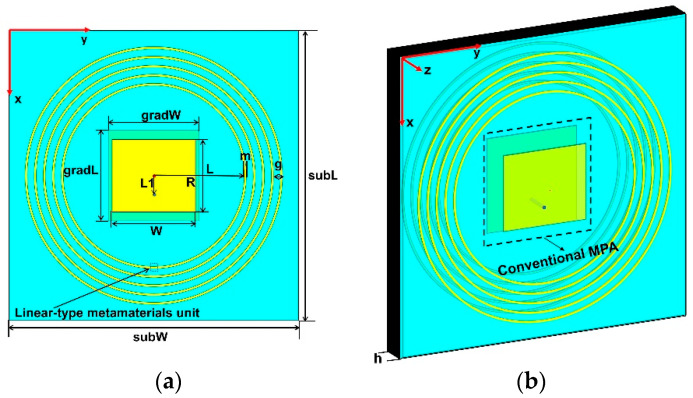
Schematic diagram of the MPA loaded with linear-type NPMMs. The blue part is the PTFE substrate, the central yellow part is the conventional MPA. Five concentric rings are formed by linear-type metamaterials units. (**a**) Top view; (**b**) side view.

**Figure 3:**
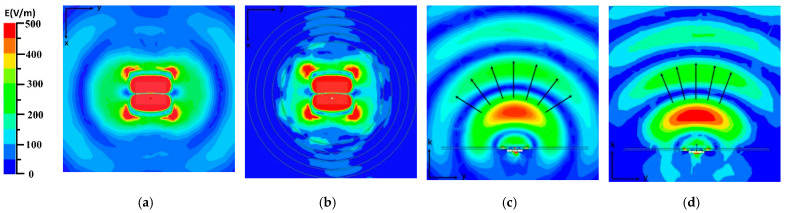
Simulated electric field distribution: (**a**,**c**) MPA in the horizontal and vertical plane; (**b**,**d**) MPA loaded with NPMMs in the horizontal and vertical plane.

The conventional MPA is composed of a radiation patch, ground plane, and PTFE plate. The detailed structure is shown in Figure 2, the parameters of the antenna are given as follows: L = 14.5 mm, W = 20 mm, gradL = 30 mm, gradW = 30 mm, subL = 115 mm, and subW = 115 mm. The antenna is fed by a 50 Ω coaxial probe with a radius of 0.65 mm along the x-direction away from the patch center, and the length of L1 is 3.4 mm. The center frequency is 5.0 GHz. The bandwidth is 230 MHz (4.89–5.12 GHz). The electric field distribution excited by a surface wave is shown in Figure 3a. According to the electric field distribution, the linear-type NPMMs units surround the patch in polar coordinates, as shown in Figure 2; the distance between the innermost metamaterials and the center of patch R is 34 mm, and its size is about λ/2. The value of R is optimized to ensure the nearest distance without coupling with the patch. The interval of the metamaterials g is 5.5 mm, and the total width of the five-loop metamaterials units is about  λ/4, which means that the surface wave passing through quarter-wavelength metamaterials can be suppressed observably. In order to ensure the electrical continuity under the limited length of the metal line, the distribution of the metamaterials is different from the x-y direction of other documents.

The electric field distribution of the MPA loaded with NPMMs in the horizontal direction is shown in Figure 3b. The energy diffraction at the boundary of the PTFE plate is obviously reduced. It means that the surface wave is well suppressed by the linear-type metamaterials. The comparison of the electric field radiation in the vertical direction of the two antennas is shown in Figure 3c,d. It can be seen that due to the linear-type NPMMs, the wave-front along the z direction tends to change from spherical wave to plane wave. Thus, the directivity of the MPA is improved. According to the law of conservation of energy, the total energy radiated by the MPA loaded with NPMMs remains unchanged, and the reduction of the lateral radiation will inevitably lead to an increase in the forward radiation. Thereby, the gain is enhanced in the main radiation direction. The maximum volume current density at the edge of the substrate along the x-axis is decreased from 0.4 to 0.04 A/m^2^, and the maximum average Poynting vector is reduced from 23.27 to 5.09 W/m^2^.

**Figure 4:**
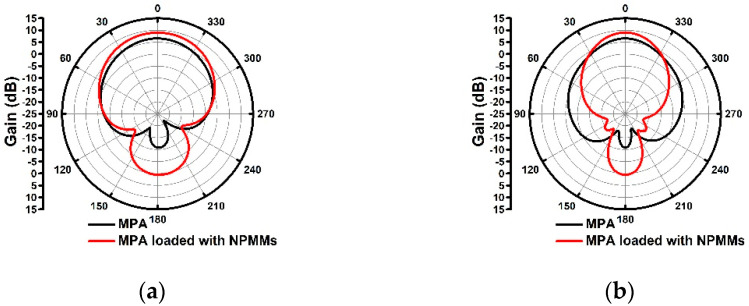
Simulated gain comparison (**a**) E-plane; (**b**) H-plane.

The center frequency and bandwidth of the MPA loaded with NPMMs are the same as the conventional MPA. The gain comparison of the two antennas at 5.0 GHz is shown in Figure 4. The gain is increased from 6.55 to 8.98 dB. The half-power beam width (HPBW) of the H-plane is reduced from 64° to 38°.

In order to verify the broadband characteristics of linear-type NPMMs, three groups of MPAs operating at the L-band, S-band, and C-band are simulated simultaneously. The substrate thickness of all antennas is 2 mm. The center frequencies are 1.5 GHz, 3.5 GHz, and 8.4 GHz, respectively. Adding the X-band antennas, Table 1 lists the main parameters of the four group antennas, including gain, directivity, and HPBW. Directivity is calculated by Kraus approximation formula $D=\frac{41253}{\theta_{E}\theta_{H}}$, where $\theta_{E}$ and ${\;\theta }_{H}$ are the HPBW of the E-plate and H-plate, respectively. It can be seen from the data that compared with the conventional antennas, the gain is improved by 1.48–2.56 dB, and the directivity is improved by 0.15–6.28. At 8.4 GHz, the inductance of the feed probe is too large, which affects the performance of this group antennas. As a result, the linear-type NPMMs do not improve the MAP at the C-band significantly. However, better gain and directivity enhancements can still be obtained by optimizing the parameters of the MPA and linear-type NPMMs unit.

## 4. Measurement Results

**Figure 5:**
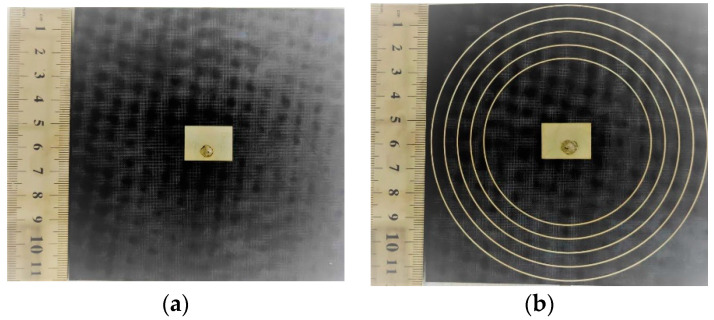
Fabricated antennas. The black part is the PTFE substrate with a permittivity of 3.5, the white part is the patch antenna and metamaterial units. The white spot in the center is the solder left by the welded SMA connector. (**a**) MPA; (**b**) MPA loaded with linear-type NPMMs.

**Figure 6:**
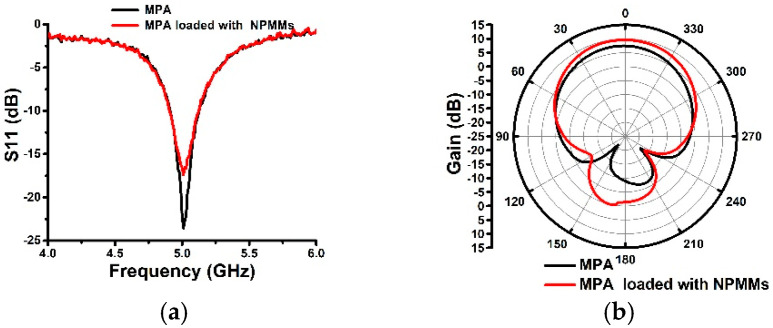

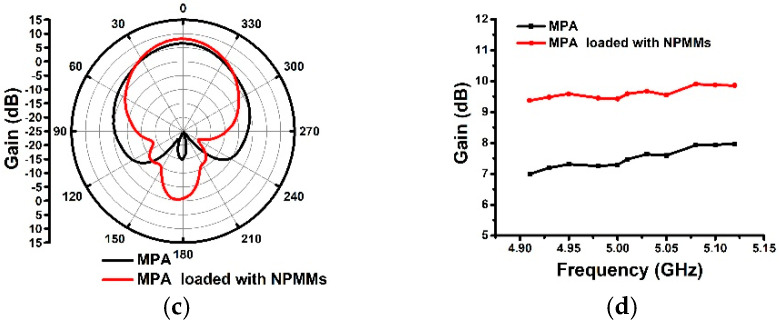
Measured parameters of the two antennas (**a**) return loss S11; (**b**) gain in the E-plane; (**c**) gain in the H-plane gain; (**d**) gains versus frequency.

A group of antennas working at the C-band are fabricated by the printed circuit board technology according to the third part simulation parameters. The antennas are fed back by a 50 Ω sub miniature version A (SMA) connector, and the fabricated antennas are shown in Figure 5. The return loss (S11) of the two antennas is measured by an integrated vector network analyzer (AV3618), as shown in Figure 6a. The center frequency of the two antennas is 5.01 GHz. The relative bandwidths of two antennas are both 4.2% from 4.91 to 5.12 GHz. The S11 trough of the conventional MPA is −22 dB, and the S11 trough of the MPA loaded with linear-type NPMMs is −18 dB. Compared with the simulation results, the center frequency moves 10 MHz to high frequency. This is mainly caused by the machining error and the uneven permittivity of the PTFE plate. The measured S11 and bandwidth are in good agreement with the simulation results, which proves that the simulated results based on HFSS are correct and reliable.

The gain of the two antennas is measured in the microwave anechoic chamber at 5.01 GHz. The radiation patterns of two antennas are shown in Figure 6b,c. The gain is changed from 7.47 to 9.58 dB with an increment of 2.12 dB.

The gain is reduced by 7.54 dB and 10.79 dB in the H-plane at 90° and 270°, respectively. The HPBW of the H-plane is reduced from 72° to 58°. The HPBW of the E-plane is reduced from 92° to 88°. Due to the linear-type metamaterials are inconsistent with the electric field distribution along the y-axis in the horizontal plane, this beam is converged with little change. Consequently, the effect of suppressing the surface wave along the y-axis is poor. However, the HPBW of the H-plane (yoz) shrinks more. According to the Friis transmission formula [7,8], the effective area can be expressed as:

(7) $$A_{e=}\frac{G}{4\pi }\lambda^{2}\;$$

where G is the gain of antenna, and  λ is wavelength. The $A_{e}$ of conventional MPA is 16 cm^2^, and the $A_{e}$ of MPA loaded with NPMMs is 26 cm^2^. Therefore, the aperture efficiency is also improved. From the above analysis, it can be drawn that the linear-type NPMMs can greatly improve the performance of MPA.

Figure 6d shows the gain comparison of 11 frequencies measured from 4.91 to 5.12 GHz. Since linear-type metamaterials have negative permeability at 1–10 GHz, when the surface wave passes through the linear-type metamaterials, a resonance forbidden gap appears. It can be seen from Figure 6d that the gain of these 11 points is increased obviously. Therefore, the surface wave can be minimized to improve the performance of MAP well.

## 5. Conclusions

In order to adopt metamaterials to improve the EM performance of UWB or multi-band microstrip antennas, this paper proposes a novel MPA based on linear-type NPMMs substrate. The simulations and measurements consistently manifest that without changing the center frequency and bandwidth of the conventional MPA, the gain is improved by 2.12 dB. In addition, the HPBW of the H-plate is reduced by 14° and the effective area is increased by 62.5%. The linear-type metamaterials have small machining error due to its simple structure. This MPA loaded with NPMMs is easy to manufacture by a printed circuit board, and it can be conveniently applied to aircraft conformal antennas, wireless energy harvesting (WEH) systems, and so on.

## Figures and Tables

**Table 1:** Simulation of the different frequency bands MPAs loaded with linear-type NPMMs.

Center Frequency			Gain		HPBW				Directivity	
	R/mm	g/mm	MPA/dB	MPA Loaded with NPMMs/dB	MPA		MPA Loaded with NPMMs		MPA	MPA Loaded with NPMMs
					$\theta_{E}$	$\theta_{H}$	$\theta_{E}$	$\theta_{H}$		
1.5 GHz	120	9	6.24	8.22	$91^{{}^{\circ}}$	$98^{{}^{\circ}}$	$74^{{}^{\circ}}$	$54^{{}^{\circ}}$	4.63	10.32
3.5 GHz	45	7	6.40	8.96	$92^{{}^{\circ}}$	$92^{{}^{\circ}}$	$74^{{}^{\circ}}$	$52^{{}^{\circ}}$	4.87	11.15
5 GHz	34	5.5	6.55	8.98	$86^{{}^{\circ}}$	$64^{{}^{\circ}}$	$83^{{}^{\circ}}$	$38^{{}^{\circ}}$	7.5	13.08
8.4 GHz	22	3	6.82	8.3	$88^{{}^{\circ}}$	$60^{{}^{\circ}}$	$96^{{}^{\circ}}$	$54^{{}^{\circ}}$	7.81	7.96

## Data Availability

The data that support the findings of this study are available from the corresponding author upon reasonable request.

## References

[1] Veselago V.G. (1968). The electrodynamics of substances with simultaneously negative values of ε and μ. Physic Uspekhi.

[2] Schurig D., Mock J.J., Justice B.J., Cummer S.A., Pendry J.B., Starr A.F., Smith D.R. (2006). Metamaterial electromagnetic cloak at microwave frequencies. Science.

[3] Landy N.I., Sajuyigbe S., Mock J.J., Smith D.R., Padilla W.J. (2008). Perfect metamaterial absorber. Phys. Rev. Lett..

[4] Asl A.B., Pourkhalil D., Rostami A., Mirtaghioglu H. (2021). A perfect electrically tunable graphene-based metamaterial absorber. J. Comput. Electron..

[5] Singh R.K., Gupta A. (2021). A wrenched-square shaped polarization independent and wide angle stable ultra-thin metamaterial absorber for s-band, x-band and ku-band applications. AEU Int. J. Electron. Commun..

[6] Zhang X.M., Liu H.X., Li L. (2017). Tri-band miniaturized wide-angle and polarization-insensitive metasurface for ambient energy harvesting. Appl. Phys. Lett..

[7] Zhong H.T., Yang X.X., Tan C., Yu K. (2016). Triple-band polarization-insensitive and wide-angle metamaterial array for electromagnetic energy harvesting. Appl. Phys. Lett..

[8] Duan X., Chen X., Zhou Y.H., Zhou L., Hao S.J. (2018). Wideband metamaterial electromagnetic energy harvester with high capture efficiency and wide incident angle. IEEE Antennas Wirel. Propag. Lett..

[9] Aldhaeebi M.A., Almoneef T.S. (2020). Planar dual polarized metasurface array for microwave energy harvesting. Electronics.

[10] Danila O., Manaila-Maximean D. (2021). Bifunctional metamaterials using spatial phase gradient architectures: Generalized reflection and refraction considerations. Materials.

[11] Danila O. (2020). Spectroscopic assessment of a simple hybrid si-Au cell metasurface-based sensor in the mid-infrared domain. J. Quant. Spectrosc. Radiat. Transf..

[12] Danila O. (2021). Polyvinylidene fluoride-based metasurface for high-quality active switching and spectrum shaping in the terahertz g-band. Polymers.

[13] James J.R., Hall P.S. (1989). Handbook of Microstrip Antennas. Handbook of Microstrip Antennas.

[14] Nasimuddin N., Chen Z.N., Qing X. (2016). Bandwidth enhancement of a single-feed circularly polarized antenna using a metasurface: Metamaterial-based wideband CP rectangular microstrip antenna. IEEE Antennas Propag. Mag..

[15] Singh A.K., Abegaonkar M.P., Koul S.K. (2017). High-gain and high-aperture-efficiency cavity resonator antenna using metamaterial superstrate. IEEE Antennas Wirel. Propag. Lett..

[16] Arora C., Pattnaik S.S., Baral R.N. (2018). Metamaterial inspired DNG superstrate for performance improvement of microstrip patch antenna array. Int. J. Microw. Wirel. Technol..

[17] Gao X., Zhang Y., Li S. (2020). High refractive index metamaterial superstrate for microstrip patch antenna performance improvement. Front. Phys..

[18] Borazjani O., Naser-Moghadasi M., Rashed-Mohassel J., Sadeghzadeh R.A. (2020). Design and fabrication of a new high gain multilayer negative refractive index metamaterial antenna for X-band applications. Int. J. RF Microw. Comput. Aided Eng..

[19] Liu Y.H., Zhao X.P. (2010). Enhanced patch antenna performances using dendritic structure metamaterials. Microw. Opt. Technol. Lett..

[20] Liu Y.H., Zhao X.P. (2010). High gain patch antenna with composite right-left handed structure and dendritic cell metamaterials. J. Infrared Millim. Terahertz Waves.

[21] Zhou B., Cui T.J. (2011). Directivity enhancement to vivaldi antennas using compactly anisotropic zero-index metamaterials. IEEE Antennas Wirel. Propag. Lett..

[22] Yang W., Wang H., Che W., Wang J. (2013). A wideband and high-gain edge-fed patch antenna and array using artificial magnetic conductor structures. IEEE Antennas Wirel. Propag. Lett..

[23] Cao W., Hong W. Bandwidth and gain enhancement for single-fed compact microstrip antenna by loading with parasitical patches. Proceedings of the IEEE International Conference on Microwave & Millimeter Wave Technology.

[24] Pendry J.B., Holden A., Stewart W., Youngs I. (1996). Extremely low frequency plasmons in metallic mesostructures. Phys. Rev. Lett..

[25] Pendry J.B., Smith D.R. (2004). Reversing light with negative refraction. Phys. Today.

[26] Smith D.R., Vier D.C., Koschny T., Soukoulis C.M. (2005). Electromagnetic parameter retrieval from inhomogeneous metamaterials. Phys. Rev. E Stat. Nonlinear Soft Matter Phys..

[27] Chen X., Grzegorczyk T.M., Wu B.I., Pacheco J., Kong J.A. (2004). Robust method to retrieve the constitutive effective parameters of metamaterials. Phys. Rev. E.

